# Introductory Editorial

**Published:** 2008-04-09

**Authors:** Gilles J. Guillemin

**Affiliations:** Head of the Neuroinflammation Group, University of New South Wales, Sydney, NSW, Australia

I am pleased to announce the launch of *International Journal of Tryptophan Research* (IJTR)—a new peer reviewed open access journal published by Libertas Academica.

The *International Journal of Tryptophan Research* is an international, open access, peer reviewed journal, which covers all subjects related to Tryptophan research. There is no suitable journal that focuses on Tryptophan, and the *IJTR* aims to fill this void and provide a forum for those with interests in this area.

The journal also covers related precursors and metabolites such as serotonin, melatonin, niacin, tryptamine, kynurenine, and studies related to enzymes such as indoleamine 2,3-dioxygenase and tryptophan hydroxylase. Topics include isolation, functionality, interactions, analysis, synthesis and industrial production, as well as supplementation and role in diet, physiology, neurological disorders and immune response. However all articles related to Tryptophan are welcome.

The journal will be competing head-on with a number of existing subscription-based journals. However, there is clearly a niche for the new journal. The reason for this is because all journal articles will be accessible without any access boundaries to all internet users throughout the world. Another major benefit of open access online journals is that anyone can contribute, and not only those in major institutions. These freedoms are coupled with rigorous, fair and prompt standards of peer review.

*International Journal of Tryptophan Research* is published exclusively online. Articles will follow a consistent format so that the visual impact will be high and equal to that of the best hard-copy publications. In contrast to paper-based journals, however, the electronic format allows the full use of digital technologies and permits the inclusion of large data sets, from field and laboratory studies, links to other web pages, animations, slide shows, video clips and unlimited colour, all at no additional charge. Open access means that all articles are freely available to all, worldwide, and at no cost to the reader. Authors retain copyright of their work and can grant anyone the right to reproduce and disseminate it, provided that it is correctly cited and no errors are introduced, under the Creative Commons “CC-BY” licence.

In hard-copy journals, the costs of publication are met by subscriptions, paid by the reader. In *International Journal of Tryptophan Research*, as in other open access journals, these costs are borne by the author in the form of a publication processing fee. Many grant-awarding bodies recognise the value of open access publishing by allowing their funds to be used for PPFs. Fee waivers and discounts are available on a case-by-case basis, and we shall make every effort to ensure that lack of funds does not impede the overall objective of publishing the best science, irrespective of authorship or country of origin.

I do not foresee that open access, online journals will totally replace the traditional print format in the immediate future, although this may be an increasing trend with time. I am certain, however, that the benefits of online publication, and the extra opportunities that digital technologies give to authors, will be increasingly recognised. Open access is of huge benefit to the researchers working in institutions around the world where institutional libraries are unable to afford subscription fees for a full range of journals.

I expect that *International Journal of Tryptophan Research* will attract manuscripts of the highest quality which are of the greatest possible benefit to readers. Peer review is undertaken by at least two leading experts in the area of the manuscript.

For further information on what we hope will be an exciting and highly useful new journal, please click on the “about this journal” links on the journal’s web page.

